# Epidemic intelligence activities among national public and animal health agencies: a European cross-sectional study

**DOI:** 10.1186/s12889-023-16396-y

**Published:** 2023-08-04

**Authors:** Timothee Dub, Henna Mäkelä, Esther Van Kleef, Agnes Leblond, Alizé Mercier, Viviane Hénaux, Fanny Bouyer, Aurelie Binot, Oumy Thiongane, Renaud Lancelot, Valentina Delconte, Lea Zamuner, Wim Van Bortel, Elena Arsevska

**Affiliations:** 1 Department of Health security, Finish Institute for Health and Welfare, Helsinki, Finland; 2 Department of Public Health, Institute of tropical medicine, Antwerp, Belgium; 3 UMR EPIA, INRAE, VetAgro Sup, University of Lyon, Marcy l’Etoile, F-69280 France; 4 Joint Research Unit Animal, Health, Territories, Risks, Ecosystems (UMR ASTRE), French Agricultural Research Centre for International Development (CIRAD), National Research Institute for Agriculture, Food and Environment (INRAE), Montpellier, France; 5 Unité Epidémiologie et appui à la surveillance, Université de Lyon–Agence nationale de sécurité sanitaire de l’alimentation, de l’environnement et du travail (Anses), Lyon, France; 6 Groupe d’Expérimentation et de Recherche: Développement et Actions Locales (GERDAL), Angers, France; 7 OpenGeoHub foundation, Agro Business Park 10, Wageningen, The Netherlands; 8 Outbreak Research Team, Department of Biomedical Sciences, Institute of tropical medicine, Antwerp, Belgium; 9 Unit of Entomology, Department of Biomedical Sciences, Institute of tropical medicine, Antwerp, Belgium

**Keywords:** Epidemic intelligence, Indicator-based surveillance, Event-based surveillance, Outbreak detection

## Abstract

Epidemic Intelligence (EI) encompasses all activities related to early identification, verification, analysis, assessment, and investigation of health threats. It integrates an indicator-based (IBS) component using systematically collected surveillance data, and an event-based component (EBS), using non-official, non-verified, non-structured data from multiple sources.

We described current EI practices in Europe by conducting a survey of national Public Health (PH) and Animal Health (AH) agencies. We included generic questions on the structure, mandate and scope of the institute, on the existence and coordination of EI activities, followed by a section where respondents provided a description of EI activities for three diseases out of seven disease models. Out of 81 gatekeeper agencies from 41 countries contacted, 34 agencies (42%) from 26 (63%) different countries responded, out of which, 32 conducted EI activities. Less than half (15/32; 47%) had teams dedicated to EI activities and 56% (18/34) had Standard Operating Procedures (SOPs) in place. On a national level, a combination of IBS and EBS was the most common data source. Most respondents monitored the epidemiological situation in bordering countries, the rest of Europe and the world. EI systems were heterogeneous across countries and diseases. National IBS activities strongly relied on mandatory laboratory-based surveillance systems. The collection, analysis and interpretation of IBS information was performed manually for most disease models. Depending on the disease, some respondents did not have any EBS activity. Most respondents conducted signal assessment manually through expert review. Cross-sectoral collaboration was heterogeneous. More than half of the responding institutes collaborated on various levels (data sharing, communication, etc.) with neighbouring countries and/or international structures, across most disease models.

Our findings emphasise a notable engagement in EI activities across PH and AH institutes of Europe, but opportunities exist for better integration, standardisation, and automatization of these efforts. A strong reliance on traditional IBS and laboratory-based surveillance systems, emphasises the key role of in-country laboratories networks. EI activities may benefit particularly from investments in cross-border collaboration, the development of methods that can automatise signal assessment in both IBS and EBS data, as well as further investments in the collection of EBS data beyond scientific literature and mainstream media.

**Supplementary Information:**

The online version contains supplementary material available at 10.1186/s12889-023-16396-y.

## Background

Epidemic Intelligence (EI) is the framework that encompasses all activities related to early identification of potential health threats, their verification, analysis, assessment, and investigation in order to recommend measures to control them. EI integrates both an indicator-based (IBS) and event-based component (EBS) [1]. IBS uses official, verified and structured data of routine surveillance systems, while EBS corresponds to the use of non-official, non-verified, non-structured data from multiple sources. In the early 2000s, following the widespread of the new pathogen causing Severe Acute Respiratory Syndrome (SARS), the public and animal health authorities (PH/AH) demand for timely information and access to data on emerging health threats increased. This led to the inception of EBS through the use of non-official sources, such as online media and expert networks [2]. It inspired the development of Internet scanning tools (e.g., the Global Public Health Intelligence Network, GPHIN), and further use of pre-existing email distribution lists, or networks (e.g., ProMED-mail) that were identified as useful data sources to complement the early warning function of routine surveillance systems.

The revised International Health Regulations (IHR) in 2005 required PH authorities to strengthen and maintain their capacity to detect, assess and report events that may constitute a health emergency of international concern [3]. As a result, in 2006, the European Centre for Disease Prevention and Control (ECDC) conducted a first assessment of the EI activities among PH agencies in Europe, showing that agencies had regular EI activities, but with various approaches and strategies. The study further noted a need for integrated rather than disease-specific EI structures and processes. In addition, this work identified a necessity for EI guidelines to be developed by the ECDC in collaboration with national health authorities in Europe [2, 4].

Since 2006, the ECDC provides the European Union (EU) PH agencies with a centralised detection, assessment, follow-up, as well as exchange of information system to improve PH surveillance and early detection of cross border and emerging infectious disease threats, offering an EI service to the Member States in addition to their national EI activities [5, 6]. Additionally, in 2021, in the midst of the COVID-19 pandemic, the World Health Organisation (WHO) set the Hub for Pandemic and Epidemic Intelligence. Its main aims are to (i) provide greater capabilities for data analytics, (ii) develop digital platforms, with access to a wider range of data sources, (iii) better foresee risk management, and (iv) have a greater focus on regional and global health threats beyond countries in the EU and European Economic Area (EEA) [7].

Fifteen years after the first assessment of EI activities among EU PH agencies and in light of the influence of climate change on infectious disease, including, but not limited to zoonosis and vector-borne diseases (VBD), emergence and distribution, there is a need to better understand how national EI systems evolved to bridge gaps in making data-driven decisions [2, 8]. In addition, while EI activities among PH agencies have been assessed, knowledge on the EI among European national AH agencies remains scarce.

As part of an effort to improve Epidemic Intelligence, the Horizon 2020 project: MOnitoring Outbreak events for Disease surveillance in a data science context (MOOD) aims at developing tools and solution to improve detection, monitoring, and assessment of emerging diseases in Europe in an integrated inter-sectorial, interdisciplinary, One health approach using big data sources in combination with environmental and socio-economic covariates. Developing these tools required a detailed knowledge of current EI activities and practices in target Public, Animal and One Health institutes.

We provide an updated overview of the current EI systems in European countries through a description of capacities and resources used in PH and AH agencies and a comparison of activities, processes and collaborations thereof.

## Methods

We conducted a large-scale online cross-sectional survey of key stakeholders at national level, responsible for conducting EI or communicable disease surveillance (further referred to as gatekeepers), from PH and AH national agencies from 42 European countries or territories: EU, EFTA, EU candidates/potential candidates, European neighbourhood policy and other European countries.

### Model pathogens

We focused on selected infectious diseases according to (i) their current impact on public and animal health in Europe; (ii) the economic cost related to their medical care and for outbreak monitoring and control; (iii) their sensitivity to climate and other environmental changes and potential to further emerge; and (iv) their representativeness of different disease systems (transmission routes).

The selected disease models were: (i) seasonal influenza and highly pathogenic avian influenza (HPAI) for airborne pathogens [9, 10]; (ii) tick-borne encephalitis (TBE) and Lyme borreliosis (LB) as models of endemic pathogens transmitted by endemic vectors [11]; (iii) West Nile virus (WNV) as an example of arbovirus transmitted by native vectors [12]; (iv) chikungunya, dengue and Zika as models of exotic pathogens transmitted by invasive mosquito species, hereafter referred to as invasive mosquito-borne diseases (IMBD) [13]; (v) Tularaemia and Leptospirosis as models of neglected endemic pathogens with multiple transmission routes and reservoirs (ND) [14–16]; (vi) antimicrobial resistant (AMR) bacterial strains as models of complex, anthropogenic disease threats [17]; (vii) unknown pathogens (disease X) as a challenge for any EI system, represented through SARS-CoV-2.

### Type and structure of the questionnaire

We invited gatekeepers via email to fill out a semi-structured survey (Supplementary material 1) regarding their agencies’ EI activities, incorporating input from relevant co-workers in charge of specific diseases if needed. We used EUsurvey, a web-based tool, that allowed for the possibility to consult or share the survey with other experts on disease surveillance so that they could save their answers and go back to the survey.

The survey consisted of three different sections following the commonly accepted framework of EI as described in 2006 by Paquet et al. [1, 2, 4, 18, 19]. The first section included generic questions on the structure, mandate (Public, Animal and/or One Health) and scope (infectious diseases under surveillance) of the respondent’s institute, on the existence, coordination, and output of the EI activities at national level, and personnel involved.

The second section was disease specific. Respondents were instructed to choose three model diseases and to provide a detailed description of the IBS and EBS EI activities related to each of these three diseases of choice. The information collected covered the following areas: data sources, workflow for detection, verification and assessment of signals, communication of alerts, and finally, international and intersectoral collaborations.

The third section of the survey allowed the respondent to indicate if the EI activities of the remaining surveyed diseases were identical to the ones they had already answered for. At the end of the survey, the respondents were provided a space for free comments to complete missing information.

All respondents were informed about the purpose of the study and consented to participate.

## Results

### Existence and organisation of the Epidemic intelligence activities

**Fig. 1:**
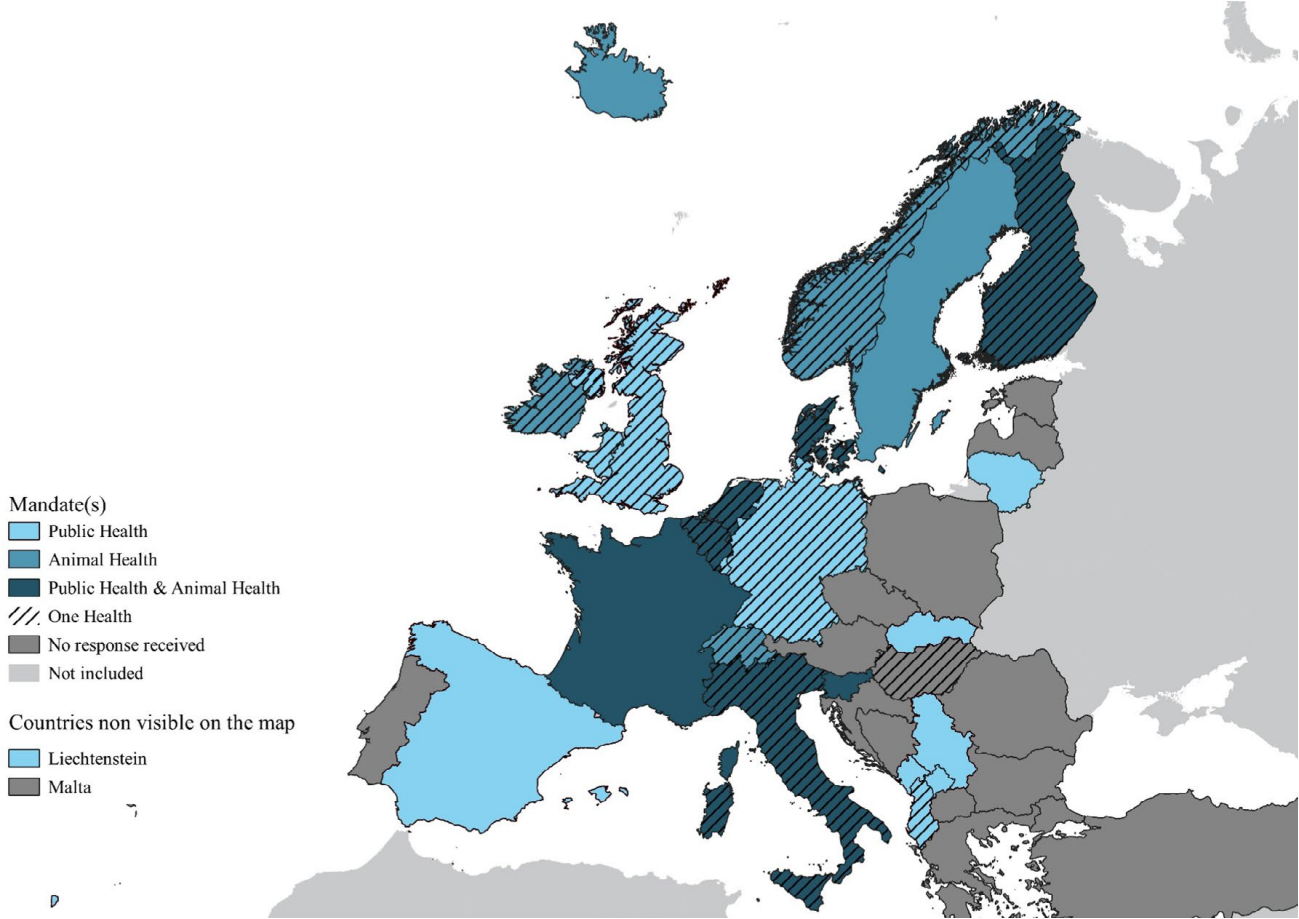
Countries and mandate of responding institutes, as of March 2021

The online survey was open from 18 February 2021 to 18 April 2021. Out of 81 PH or AH gatekeeper agencies contacted from 41 different countries or territories, 34 agencies (42%) from 26 (63%) different countries or territories responded (Fig. 1). When asked about their primary mandates, 61% of agencies identified themselves as having a PH (n = 21/34), 56% an AH (19/34) and 53% a One Health (OH) (18/34) mandate. Additionally, three respondents (9%) indicated that they had a food safety mandate and two respondents (6%) had in addition a research mandate. Out of the 34 respondents, 65% had more than one single mandate (22/34).

The majority of agencies monitored animal infectious diseases (*n* = 22; 65%), human infectious diseases (*n* = 18; 53%) and food products of animal origin (*n* = 17; 50%). Twelve agencies reported they monitored both human and animal infectious diseases (12/34, 35%).

Out of 34 respondents from 26 countries, 32 from 24 different countries performed EI activities (Supplementary Table 1). Among them, the number of full-time equivalent employees (FTE) engaged in EI activities ranged from only one FTE for 19% of the respondents (6/32) to more than ten FTE for 29% of the respondents (9/32). However, less than half (15/32; 47%) of the agencies performing EI activities had dedicated teams.

The majority of the respondents stated the existence of Standard Operating Procedures (SOPs) for EI (18/34; 56%); in eleven agencies the SOPs were publicly available (11/18; 61%). All SOPs covered the detection of signals of potential health hazards related to infectious disease outbreak (18/18, 100%), followed by signal communication (15/18; 83%) and signal assessment (14/18; 78%). Filtering and verification of signals were covered by only 10/18 (56%) and 12/18 (67%) SOPs, respectively.

### Diseases described

The majority of respondents described their EI activities for SARS-CoV-2 (*n* = 20), either in humans (*n* = 12) or in animals (*n* = 8), followed by HPAI in animals (*n* = 19) and seasonal influenza (in humans; *n* = 10). Surveillance of TBE was only described by two agencies, while LB and AMR surveillance in humans were chosen by a single respondent, hence not reported in our findings (Supplementary Table 2).

### Epidemic intelligence activities

Data sources for EI varied depending on the country, disease in question and geographical area monitored. On a national level, a combination of IBS and EBS was the most common source of information for EI (Table 1), and notably for IMBD in humans (5/6 respondents, 83%), WNV in humans and animals (7/9 respondents, 78% and 4/7 respondents, 57%, respectively), SARS-CoV-2 in humans and animals (8/11 respondents, 73% and 5/8 respondents, 63%, respectively) and AMR in animals (4/6 respondents, 67%). IBS was the major source of information for Tularaemia and Leptospirosis in animals on a national level (4/7 respondents, 57%).

Most respondents also conducted EI activities by monitoring the epidemiological situation in the bordering countries and/or the rest of Europe, using a variety of sources. A combination of IBS and EBS was predominantly reported for WNV in humans (5/9 respondents, 56%) both in the bordering countries and in Europe, for SARS-CoV-2 in animals (2/3 respondents, 67%) in the bordering countries and SARS-CoV-2 in humans (7/10 respondents, 70%) in Europe, however, two out of the three (67%) respondents who provided answers on the monitoring of epidemiological situation regarding ND in animals only used EBS (Table 1). The number of agencies that were not monitoring the epidemiological situation in bordering countries and/or the rest of Europe was low: three out of ten respondents for seasonal influenza, followed by two out of fourteen agencies responding for HPAI (14%), and one out of seven of the agencies who described WNV in animals surveillance activities (14%).

A diversity of sources was also used for monitoring EI worldwide, with a notable combination of IBS and EBS for Leptospirosis and Tularaemia (2/3 respondents, 67%), WNV in animals (3/5 respondents, 60%) and SARS-CoV-2 in humans (6/11 respondents, 55%). All respondents reported they monitored the epidemiological situation worldwide for seasonal influenza, IMBD in humans, AMR in animals and SARS-CoV-2 in animals. The proportion of respondents that did not monitor the worldwide epidemiological situation ranged from 1/11 (9%) respondents for SARS-CoV-2 in humans to 2/5 (40%) respondents for WNV in animals.

**Table 1:** Epidemic intelligence (EI) activities per diseases described by responding institutes conducting EI, as of March 2021

	HPAI in animals (*n* = 17)	Seasonal Influenza (*n* = 10)	WNV in animals (*n* = 7)	WNV in humans (*n* = 9)	Leptospirosis and Tularemia in animals (*n* = 7)	Invasive MBD in humans (*n* = 6)	AMR in animals (*N* = 6)	SARS-COV-2 in humans (*n* = 12)	SARS-CoV-2 in animals (*n* = 8)
**Nationally**									
Indicator based surveillance only	8 (47%)	4 (40%)	2 (29%)	2 (22%)	4 (57%)	1 (17%)	2 (33%)	3 (27%)	3 (37.5%)
Event-based surveillance only	0	0	1 (14%)	0	0	0	0	0	0
IBS and EBS	9 (53%)	6 (60%)	4 (57%)	7 (78%)	3 (43%)	5 (83%)	4 (67%)	8 (73%)	5 (62.5%)
No surveillance activities	0	0	0	0	0	0	0	0	0
**Bordering countries**									
Indicator based surveillance only	3 (23%)	1 (10%)	0	2 (22%)	0	1 (17%)	1 (25%)	1 (10%)	0
Event-based surveillance only	4 (31%)	2 (20%)	2 (40%)	2 (22%)	2 (67%)	2 (33%)	1 (25%)	3 (30%)	1 (33%)
IBS and EBS	4 (31%)	4 (40%)	2 (40%)	5 (56%)	1 (33%)	3 (50%)	2 (50%)	5 (50%)	2 (67%)
No surveillance activities	2 (15%)	3 (30%)	1 (20%)	0	0	0	0	1 (10%)	0
**Europe**									
Indicator based surveillance only	4 (29%)	3 (33%)	1 (14%)	2 (22%)	0	1 (17%)	1 (25%)	1 (10%)	1 (25%)
Event-based surveillance only	4 (29%)	2 (22%)	2 (29%)	2 (22%)	2 (67%)	2 (33%)	1 (25%)	2 (20%)	1 (25%)
IBS and EBS	4 (29%)	4 (44%)	3 (43%)	5 (56%)	1 (33%)	3 (50%)	2 (50%)	7 (70%)	2 (50%)
No surveillance activities	2 (14%)	0	1 (14%)	0	0	0	0	0	0
**Worldwide**									
Indicator based surveillance only	3 (21%)	3 (33%)	0	0	0	1 (17%)	1 (25%)	1 (9%)	0
Event-based surveillance only	4 (29%)	2 (22%)	0	3 (38%)	0	2 (33%)	1 (25%)	3 (27%)	1 (33%)
IBS and EBS	5 (36%)	4 (44%)	3 (60%)	4 (50%)	2 (67%)	3 (50%)	2 (50%)	6 (55%)	2 (67%)
No surveillance activities	2 (14%)	0	2 (40%)	1 (13%)	1 (33%)	0	0	1 (9%)	0

#### Indicator-based activities: data sources and processes

IBS activities strongly relied on mandatory laboratory-based surveillance systems for the majority of respondents, across all diseases, notably among all respondents for HPAI (17/17), WNV in humans (9/9), and IMBD in humans (6/6), while for other diseases, the use of mandatory laboratory-based surveillance ranged from 57 to 83% of the respondents. For only one disease model, i.e., seasonal influenza, the use of sentinel laboratory-based surveillance was more common, reported by all respondents (10/10 respondents, 100%) compared to laboratory based mandatory surveillance reported by seven agencies (7/10 respondents, 70%) (Table 2).

The use of syndromic surveillance was heterogeneous depending on the respondent and model disease. The use of mandatory and sentinel syndromic surveillance was highest for seasonal influenza, with 6/10 and 7/10 respondents, respectively. Mandatory syndromic surveillance was not reported for WNV surveillance in humans (0/9 respondents) and ND (0/10 respondents). None of the respondents conducted syndromic surveillance, either mandatory or sentinel for IMBD in humans (0/6 respondents) and AMR in animals (0/6 respondents).

The use of official public websites (WHO, OIE, ECDC) was common, ranging from 29% (2/7 respondents) for ND in animals to 83% (10/12 respondents) of the respondents for SARS-CoV-2 in humans. The use of official international notifications (WHO, ADNS) was also common and ranged from 29% (2/7 respondents) for ND in animals to 8/10 of the respondents for seasonal influenza.

The collection, analysis and interpretation of IBS information was performed manually for most diseases, ranging from 25% of the respondents (3/12 respondents) for SARS-CoV-2 surveillance in humans to 71% (5/7 respondents) of the respondents for WNV in animals. The use of semi-automatic methods was more common than manual methods for seasonal influenza surveillance (9/10 respondents, 90%), SARS-CoV-2 surveillance in humans (8/12 respondents, 67%) and AMR surveillance in animals (3/6 respondents, 50%). Across all diseases, only one respondent reported the use of automatic methods for collection, analysis, and interpretation of IBS data for SARS-CoV-2 surveillance in humans.

#### Event-based activities: data sources and processes

Depending on the diseases, some respondents reported not to use any EBS sources, ranging from 17% (1/6 respondents) for IMBD to 57% respondents (4/7 respondents) for surveillance of ND in animals (Table 2). The most commonly used source of EBS data was scientific literature, reported by 10/12 (83%), 5/6 (83%) and 8/10 (80%) respondents for SARS-CoV-2 in humans, IMBD in humans and seasonal influenza in humans, respectively. All respondents reported to use scientific literature. Apart for Leptospirosis and Tularaemia (3/7 respondents, 43%), half or more respondents used mainstream media as a source of EBS data for the surveillance of each disease. The use of social media and blogs was not reported by respondents for WNV in animals, ND in animals and AMR surveillance in animals; however, this informal source was used by 67% respondents for SARS-CoV-2 surveillance in humans (8/12 respondents) and IMBD in humans (4/6 respondents). The use of specialized internet sources (ProMED, Healthmap, Gideon, etc.) was reported by 75% or more of respondents for seasonal influenza surveillance in humans (8/10, 80%), WNV in humans (7/9 respondents, 78%), IMBD in humans (5/6 respondents, 83%) and SARS-CoV-2 in humans (10/12, 83%), and by 50% or more respondents across all diseases. For ND in animals, the use of specialised internet sources was used by only 1/7 of the respondents (14%). The proportion of respondents who reported using official international notification was the same as the one using specialised internet sources for HPAI (8/17 respondents, 47%), seasonal influenza surveillance in humans (8/10 respondents, 80%), WNV in humans (7/9, 78%), IMBD in humans (5/6 respondents, 83%) and SARS-CoV-2 surveillance in humans (10/12, 83%).

Half or more of respondents reported using EI surveillance systems for seasonal influenza surveillance (6/10 respondents, 60%), WNV in humans (5/9 respondents, 56%), SARS-CoV-2 in humans (7/12, 58%) and IMBD in humans (3/6 respondents, 50%).

For several disease models, some respondents reported they did not proceed to capture, filtering and validation of EBS signals, ranging from 4/7 (57%) for ND to 17% of respondents for SARS-Cov-2 in humans (2/12) and IMBD in humans (1/6 respondents). Across all disease models, only one respondent reported using automatic methods, in the case of HPAI surveillance. The use of manual methods was twice more frequent than the use of semi-automatic methods, apart for SARS-CoV-2 surveillance in animals with 38% (3/8 respondents) using manual methods versus 25% (2/8 respondents) using semi-automatic methods.

### Signal assessment and communication of alerts

Across all disease models, when asked whether they used manual or semi-automatic methods for assessment of EBS and IBS signals, vast majority of respondents answered they conducted it manually through expert review, ranging from 50% (5/10 respondents) for seasonal influenza in humans up to all of the respondents (7/7 respondents) for WNV surveillance in animals (Table 2).

On a national level, restricted-access communication was reported by more than 80% respondents across all disease models, except for SARS-CoV-2 in animals and HPAI, where it remained high, but only reported by 63% (5/8) and 71% (12/14) respondents, respectively. Half or more respondents reported unrestricted communication of potential infectious diseases related hazards to the general public across all disease models (Table 2).

Across all diseases models, restricted access communication on international level was less common than on national level, with a maximum of 70% (7/10) respondents for seasonal influenza, and lowest reporting in animal surveillance, with 1/8 respondents (13%) in SARS-CoV-2 in animals and 1/7 respondents (14%) for ND and AMR in animals.

**Table 2:** Data sources and processes per diseases described by responding institutes conducting Epidemic Intelligence, as of March 2021

	HPAI in animals (*n* = 17)	Seasonal influenza (*n* = 10)	WNV in animals (*n* = 7)	WNV in humans (*N* = 9)	Leptospirosis and Tularemia in animals (*n* = 7)	Invasive MBD in humans (*n* = 6)	AMR in animals (*n* = 6)	SARS-CoV-2 in humans (*n* = 12)	SARS-CoV-2 in animals (*n* = 8)
**Data sources used for IBS activities**^a^									
Mandatory laboratory-based	17 (100%)	7 (70%)	5 (71%)	9 (100%)	4 (57%)	6 (100%)	5 (83%)	10 (83%)	6 (75%)
Sentinel laboratory-based	5 (29%)	10 (100%)	2 (29%)	2 (22%)	4 (57%)	2 (33%)	4 (67%)	5 (42%)	2 (25%)
Mandatory syndromic	10 (59%)	6 (60%)	1 (14%)	0	0	0	0	3 (25%)	2 (25%)
Sentinel syndromic	2 (12%)	7 (70%)	2 (29%)	1 (11%)	1 (14%)	0	0	5 (42%)	1 (12.5%)
Official public websites (WHO, OIE, ECDC)	12 (71%)	8 (80%)	3 (43%)	6 (67%)	2 (29%)	4 (67%)	2 (33%)	10 (83%)	5 (62.5%)
Official international notifications (WHO, ADNS)	13 (76%)	8 (80%)	4 (57%)	6 (67%)	2 (29%)	4 (67%)	2 (33%)	10 (83%)	6 (75%)
Other IBS sources	3 (18%)	6 (60%)	0	3 (33%)	0	1 (17%)	1 (17%)	9 (75%)	2 (25%)
No use of IBS sources	0	0	1 (14%)	0	0	0	0	0	0
**Collection, analysis and interpretation of IBS information**^a^									
Manually	11 (65%)	4 (40%)	5 (71%)	6 (67%)	5 (71%)	3 (50%)	3 (50%)	3 (25%)	5 (62.5%)
Semi-automatically	7 (41%)	9 (90%)	1 (14%)	2 (22%)	2 (29%)	2 (33%)	3 (50%)	8 (67%)	3 (37.5%)
Automatically	0	0	0	0	0	0	0	1 (8%)	0
No collection, analysis and interpretation of IBS information reported	0	0	1 (14%)	2 (22%)	0	1 (17%)	0	2 (17%)	0
**Data sources for EBS activities**^a^									
Scientific literature	8 (47%)	8 (80%)	5 (71%)	6 (67%)	3 (43%)	5 (83%)	3 (50%)	10 (83%)	5 (62.5%)
Mainstream media (newspapers etc.)	8 (47%)	6 (60%)	4 (57%)	5 (56%)	3 (43%)	4 (67%)	3 (50%)	10 (83%)	5 (62.5%)
Social media and/or blogs	2 (12%)	4 (40%)	0	5 (56%)	0	4 (67%)	0	8 (67%)	1 (12.5%)
Specialized internet sources (ProMED, Healthmap, Gideon, etc.)	8 (47%)	8 (80%)	4 (57%)	7 (78%)	1 (14%)	5 (83%)	3 (50%)	10 (83%)	4 (50%)
Official international notifications (WHO, EPIS)	8 (47%)	8 (80%)	5 (71%)	7 (78%)	2 (29%)	5 (83%)	4 (67%)	10 (83%)	5 (62.5%)
Epidemic intelligence surveillance system (EIOS)	1 (6%)	6 (60%)	1 (14%)	5 (56%)	0	3 (50%)	2 (33%)	7 (58%)	0
Other EBS sources	2 (12%)	3 (30%)	0	1 (11%)	1 (14%)	1 (17%)	0	4 (33%)	1 (12.5%)
No use of EBS sources	7 (41%)	2 (20%)	2 (29%)	2 (22%)	4 (57%)	1 (17%)	2 (33%)	2 (17%)	3 (37.5%)
**Capture, filtering and verification of EBS signals**^a^									
Manually	6 (35%)	7 (70%)	4 (57%)	6 (67%)	3 (43%)	3 (50%)	4 (67%)	8 (67%)	3 (37.5%)
Semi-automatically	3 (18%)	3 (30%)	1 (14%)	2 (22%)	0	2 (33%)	0	4 (33%)	2 (25%)
Automatically	1 (6%)	0	0	0	0	0	0	0	0
No capture, filtering and verification of EBS signals reported	7 (41%)	2 (20%)	2 (29%)	2 (22%)	4 (57%)	1 (17%)	2 (33%)	2 (17%)	3 (37.5%)
**Assessment of EBS and IBS signals**^a^									
Semi-automatically	5 (29%)	5 (50%)	0	1 (13%)	1 (14%)	2 (33%)	2 (33%)	3 (25%)	1 (12.5%)
Manually	12 (71%)	5 (50%)	7 (100%)	7 (88%)	6 (86%)	4 (67%)	4 (67%)	9 (75%)	7 (87.5%)
**Communication of alerts**^a^									
Unrestricted to the general public	14 (82%)	7 (70%)	5 (71%)	4 (50%)	5 (71%)	3 (50%)	4 (67%)	7 (58%)	4 (50%)
Restricted-access communication on a national level	12 (71%)	9 (90%)	6 (86%)	8 (100%)	6 (86%)	6 (100%)	5 (83%)	11 (92%)	5 (62.5%)
Restricted-access communication on an international level	9 (53%)	7 (70%)	1 (14%)	4 (50%)	1 (14%)	3 (50%)	2 (33%)	7 (58%)	1 (12.5%)

^a^Non mutually exclusive

### Existing collaborations

#### Cross-sectoral

Cross-sectoral collaboration was heterogeneous depending on the disease model. Less than half of the respondents reported a collaboration with other sectors for ND (2/7 respondents, 29%), WNV in animals (3/7 respondents, 43%) and SARS-CoV-2 in humans (5/12 respondents, 45%), while three quarters or more of the respondents collaborated with other sectors for WNV in humans (6/8 respondents, 75%) and IMBD in humans (5/6 respondents, 83%) (Table 3).

The subject of cross-sectoral collaboration also depended on the disease model. Sharing of surveillance results was the most commonly reported area, and notably for HPAI (11/17 respondents,75.65%), WNV in humans (6/8 respondents, 75%), IMBDs in humans (4/6, 67%) and SARS-CoV-2 in animals (5/8, 62.5%). Collaboration regarding surveillance design, data collection and data management/storage was below 40% across all disease models.

#### Collaboration with neighbouring countries and international structures

More than half responding institutes collaborated with neighbouring countries and/or international structures across all disease models, apart from WNV in animals with only 2/7 respondents reporting collaborations. SARS-CoV-2 in humans, seasonal influenza in humans, and HPAI were the disease models where collaboration with neighbouring countries and/or international structures were the most common with 11/12 (92%), 9/10 (90%) and 15/17 (88%) respondents, respectively. All responding institutes, however, collaborated as much or more with international structures than with neighbouring countries.

Regarding collaboration with international structures, data sharing ranged from 14% of the respondents for WNV in animals (1/7) to half or more for seasonal influenza (5/10 respondents, 50%), HPAI (13/17, 76%), WNV in humans (4/7 respondents, 57%), IMBD in humans (4/6 respondents, 67%). Collaboration regarding surveillance design was reported by half or more respondents for HPAI (9/17 respondents, 53%) and seasonal influenza (5/10 respondents, 50%). None of the respondents for WNV in humans reported collaboration regarding surveillance design, while for WNV in animals none of the respondents collaborated with international structures regarding data collection, data management or/and storage and data analysis and interpretation.

**Table 3:** Regional, international and intersectoral collaborations per diseases described by responding institutes conducting Epidemic Intelligence, as of March 2021

	HPAI in animals (*n* = 17)	Seasonal influenza (*n* = 10)	WNV in animals (*n* = 7)	WNV in humans (*N* = 9)	Leptospirosis and Tularemia in animals (*n* = 7)	Invasive MBD in humans (*n* = 6)	AMR in animals (*n* = 6)	SARS-CoV-2 in humans (*N* = 12)	SARS-CoV-2 in animals (*N* = 8)
**Collaboration with neighboring countries or international structures**									
No	2 (12%)	1 (10%)	5 (71%)	2 (29%)	3 (43%)	1 (17%)	3 (50%)	1 (8%)	4 (50%)
Yes	15 (88%)	9 (90%)	2 (29%)	5 (71%)	4 (57%)	5 (83%)	3 (50%)	11 (92%)	4 (50%)
**With neighboring countries**	10 (59%)	5 (50%)	1 (14%)	2 (29%)	2 (29%)	2 (33%)	1 (17%)	7 (58%)	2 (25%)
Surveillance design	4 (24%)	1 (10%)	0	0	0	1 (17%)	1 (17%)	1 (8%)	1 (12.5%)
Data collection	3 (18%)	0	0	0	0	1 (17%)	0	0	0
Data sharing	6 (35%)	3 (30%)	0	1 (14%)	0	2 (33%)	0	3 (25%)	0
Sharing of surveillance results	8 (47%)	4 (40%)	1 (14%)	1 (14%)	1 (14%)	1 (17%)	1 (17%)	3 (25%)	0
Data management or/and storage	1 (6%)	0	0	0	0	0	0	0	0
Data analysis and interpretation	3 (18%)	1 (10%)	0	0	0	1 (17%)	0	1 (8%)	0
Communication	7 (41%)	4 (40%)	1 (14%)	1 (14%)	2 (29%)	1 (17%)	0	6 (50%)	1 (12.5%)
**With international structures**	15 (88%)	9 (90%)	2 (29%)	4 (57%)	3 (43%)	4 (67%)	2 (33%)	7 (58%)	4 (50%)
Surveillance design	9 (53%)	5 (50%)	1 (14%)	0	1 (14%)	1 (17%)	1 (17%)	2 (17%)	3 (37.5%)
Data collection	7 (41%)	5 (50%)	0	1 (14%)	1 (14%)	2 (33%)	0	4 (33%)	1 (12.5%)
Data sharing	13 (76%)	5 (50%)	1 (14%)	4 (57%)	2 (29%)	4 (67%)	1 (17%)	4 (33%)	3 (37.5%)
Sharing of surveillance results	12 (71%)	8 (80%)	2 (29%)	3 (43%)	3 (43%)	3 (50%)	2 (33%)	4 (33%)	2 (25%)
Data management or/and storage	3 (18%)	4 (40%)	0	2 (29%)	0	1 (17%)	0	3 (25%)	1 (12.5%)
Data analysis and interpretation	4 (24%)	7 (70%)	0	3 (43%)	2 (29%)	3 (50%)	1 (17%)	4 (33%)	1 (12.5%)
Communication	10 (59%)	8 (80%)	2 (29%)	3 (43%)	2 (29%)	3 (50%)	1 (17%)	5 (42%)	2 (25%)
**One health, public health and animal health EI collaboration**									
No	5 (29%)	5 (50%)	4 (57%)	2 (25%)	5 (71%)	1 (17%)	2 (33%)	6 (55%)	3 (37.5%)
Yes	12 (71%)	5 (50%)	3 (43%)	6 (75%)	2 (29%)	5 (83%)	4 (67%)	5 (45%)	5 (62.5%)
**If One health, public health and animal health EI collaboration**									
Surveillance design	1 (6%)	2 (20%)	2 (29%)	1 (13%)	0	2 (33%)	1 (17%)	3 (25%)	3 (37.5%)
Data collection	3 (18%)	2 (20%)	2 (29%)	0	0	1 (17%)	1 (17%)	3 (25%)	2 (25%)
Data sharing	8 (47%)	4 (40%)	2 (29%)	3 (38%)	0	3 (50%)	2 (33%)	3 (25%)	4 (50%)
Sharing of surveillance results	11 (65%)	4 (40%)	2 (29%)	6 (75%)	1 (14%)	4 (67%)	3 (50%)	5 (42%)	5 (62.5%)
Data management or/and storage	2 (12%)	3 (30%)	2 (29%)	0	0	1 (17%)	1 (17%)	2 (17%)	0
Data analysis and interpretation	3 (18%)	3 (30%)	1 (14%)	3 (38%)	0	3 (50%)	2 (33%)	4 (33%)	2 (25%)
Communication	10 (59%)	5 (50%)	2 (29%)	4 (50%)	1 (14%)	4 (67%)	3 (50%)	4 (33%)	3 (37.5%)

## Discussion

We provide an in-depth description of the best practices in EI activities among European PH/AH/OH agencies focusing on a selection of priority diseases, useful for informing efforts related to new EI initiatives, such as the WHO pandemic Hub efforts to strengthen epidemic intelligence capacities globally and create a global surveillance ecosystem, the European Commission’s objective to support numeric innovation in EI and current EI activities conducted by the ECDC.

### Existence and organisation of epidemic intelligence activities

In line with Kaiser & Coulombier’s findings in 2006, our results showed that almost all responding institutes (32/34) collected EI data regularly on both national and international levels. Even though they had identified a need for integrated surveillance across diseases and countries, rather than disease specific EI systems, our work showed that EI activities still strongly differ between countries and are largely disease specific. We also found that almost half (15/32; 47%) of the responding agencies carrying EI had dedicated teams showing a strong progress in engagement in EI activities on a national level, compared to 2006 when only three of the 23 surveyed countries reported they had a dedicated EI team. We did not see an increase in the proportion of respondents having SOPs in place for some of the EI components, with 18/32 (56%) respondents in 2021 compared to 15/23 (65%) in 2006. However, as the existence of SOPs and guidance are important requirements for optimal and effective performance of EI surveillance systems, their lack shows a need to further strengthen capacities of European national EI systems [20–22].

### Epidemic intelligence activities

#### Indicator-based activities: data sources and processes

Across all diseases, mandatory laboratory-based surveillance systems were the main IBS source, showing the key role of in-country microbiological laboratories networks and capacities. Second, came syndromic surveillance systems and the use of official public websites (WHO, OIE, ECDC) and official international notifications (WHO, ADNS). The latter highlights the importance of timely reporting and the coordination/sharing of surveillance indicators and results to international structures [23]. The collection, analysis and interpretation of IBS information was performed manually for most diseases and across all respondents, whilst only one respondent reported the use of automatic methods for collection, analysis, and interpretation of IBS data. The absence of an automated process for this activity requires permanent availability of skilled human resources. Future works should investigate how this gap can be filled.

#### Event-based activities: data sources and processes

The use of EBS was not generalised to all disease models and countries, and when conducting EBS activities, most respondents relied on scientific literature and mainstream media. The uptake of EI systems, such as EIOS, was far from being generalised across all infectious diseases models. The generalisation of EI systems might be hampered by their limited performance for filtering noise and irrelevant items and validation of signals and analysis [24]. This requires time-consuming involvement of trained personnel, and leading agencies to favour the use of more trustworthy sources, such as scientific literature, despite poor timeliness, and mainstream media, despite limited coverage of a range of online sources. For several disease models, the capture, filtering and validation of EBS signals were not part of the EI activities, raising concerns about countries capacities to early detect potential health threats, and the need to train PH and AH professionals in collection, analysis and interpretation of EBS signals, either through in-country initiatives, European capacity building, or within the development of the WHO’s Pandemic Hub in the coming years [7, 25, 26].

### Signal assessment and communication of alerts

Our findings showed that manual assessment of IBS and EBS signals i.e., expert review, was the main method used, emphasising the demand for continuous availability of trained personnel. The implementation of automatic or semi-automatic methods and tools [19, 24, 27], including forecasting or the use of data science and artificial intelligence techniques for rapid processing of EI data could be of interest for better allocation of resources, to decrease the current workload of EI officers and effectively support government agencies, healthcare service providers, and medical professionals in the future [28, 29]. However, the implementation of such tools would require important investment in technical infrastructure, as well as training, and would, in the current setting, still require dedicated human resources for signal validation and risk assessment.

### Communication of alerts and collaborations

Communication of signals and alerts was heterogeneous; however, across all disease models, responding institutes were more commonly using restricted access communication on a national level than on international level, showing a potential lack and delay in international collaboration for early warning and response to potential PH/AH threats. Yet, when asked about ongoing EI collaborations with neighbouring countries and international structures, it appeared that most responding institutes had more collaborations with international agencies than with neighbouring countries, further highlighting the lack of regional coordination between countries.

Enhancing cross-border and international collaborations in order to tackle under-reporting and increase early detection, warning and response should be at the centre of future initiatives, taking into account disease- (severity, mode of transmission) and country-specific (public health infrastructure, epidemic preparedness, politics) factors previously identified by evaluations of the sensitivity of international epidemic intelligence tools [27, 30].

### One health, public health, and animal health EI collaboration

Even though One Health and intersectoral collaborations are widely recognized approaches to tackle infectious diseases threats, prevent emergence and provide early response to such threats [31–35], we identified that intersectoral collaboration was not the rule for most disease models, and that in most cases it was limited to sharing of surveillance results. This highlights the need to better understand the stakeholders’ expectations, levers, and barriers to intersectoral collaborations [36, 37]. A further assessment of One Health collaborations and capacities in EI should support the identification of current gaps, in a context where climate change further increases the risk of spread of zoonosis and VBD and vulnerability of Health systems, including in Europe [38, 39].

### Strengths & limitations

Our work has several limitations: First, the response rate was limited to 42% with 34 responding PH or AH agencies out of 81 gatekeepers/agencies contacted; however, respondents were well distributed over Europe, providing, we believe, a global overview of EI activities in Europe. Second, it is likely that when giving respondents the possibility to pick three diseases models, they focused on the ones with the most established EI systems and might not be representatives of potential gaps in their EI systems. For that reason, our work should be considered as an assessment of best practices in EI in Europe rather than a complete overview. Regarding accuracy of answers, by giving the responding gatekeeper the possibility to pause and resume the survey, we ensured that they could consult with colleagues and substance experts in order to improve or correct their answers, however, as mentioned above, it is also possible that respondents answered for diseases with the clearest or most established EI systems in their countries. Finally, we conducted this assessment following an established and recognized theoretical framework, hence, producing results comparable to previous assessment of PH agencies and that could be replicated in the future.

## Conclusion

Our work provides a global overview of best practices of EI activities in Europe, including for AH which was previously scarce. We identified that EI systems were heterogeneous in terms of surveillance activities and data sources and that most EI activities were conducted manually, showing a need for better integration, standardisation, and automatization of EI activities across Europe, as well as training of professionals and development of SOPs. The role of microbiological diagnostics and reference laboratories in IBS remains major across all disease systems, emphasising the need to maintain current infrastructure, while the uptake of EBS was limited overall with very few data sources used. Scientific literature was mostly consulted, despite poor timeliness as well as mainstream media, despite limited coverage of online sources. Overall, most EI activities were conducted manually, further emphasising the need to pursue the development of semi- or fully automatized EBS tools targeted at the early detection, monitoring and assessment of health threats and diseases emergence.

### Supplementary Information

**Additional file 1: Supplementary Material 1.** Online questionnaire.

**Additional file 2: Supplementary Table 1.** Disease specific epidemic intelligence activities described per countries. * Responses provided by 2 gatekeeping agencies. **Supplementary Table 2.** Characteristics of respondents per diseases described. * Non mutually exclusive.

## Data Availability

The datasets generated and/or analysed during the current study are not publicly available due to the country names and agencies they contain; but anonymized data are available from the corresponding author on reasonable request.
