# Supplementary material for: Epidemic intelligence activities among national public and animal health agencies: a European cross-sectional study
Source: BMC Public Health. 2023 Aug 4;23:1488. doi: 10.1186/s12889-023-16396-y (PMC10401758; doi:10.1186/s12889-023-16396-y)
Supplement: Supplementary file 1 — Additional file 1: Supplementary Material 1. Online questionnaire. [file 12889_2023_16396_MOESM1_ESM.pdf]

Mapping state-of-the-art Epidemic Intelligence activities in Europe

Fields marked with * are mandatory.

Mapping state-of-the-art of Epidemic Intelligence systems in Europe

Description of the project

This survey is conducted as part of the state-of-the-art activities of the Horizon 2020 MOOD-project.

The H2020 project **MOOD (Monitoring outbreak events for disease surveillance in a data science context)** aims at developing epidemic intelligence (EI) tools and services for early detection, monitoring and assessment of signals of disease emergence of importance to Europe and European national Public and Veterinary health (PH/VH) agencies.

The innovative path of MOOD is based on the integration of user-needs into the development of tools and services for EI derived from better-integrated Big data sources. This user driven development will ensure a more effective integration of the tools and services into the daily EI activities.

To develop EI tools and services, MOOD will use as case studies a selected number of disease systems of importance to Europe:

- Influenza (all virus types) for airborne pathogens;
- Tick-borne encephalitis and Lyme borreliosis as models of endemic pathogens transmitted by endemic vectors;
- West Nile and Usutu viruses as examples of exotic pathogens transmitted by endemic vectors;
- Chikungunya, dengue and Zika viruses as models of exotic pathogens transmitted by invasive mosquito species;
- Tularaemia and leptospirosis as models of neglected endemic pathogens with multiple transmission routes and reservoirs;
- Antimicrobial resistant bacterial (AMR) strains as models of complex, anthropogenic disease threats;
- Unknown pathogens (disease X), Including SARS-CoV-2, as a challenge for any EI system.

Visit the Horizon2020 MOOD-project website

Objectives of the survey

The objective of this survey is to understand the current Epidemic Intelligence (EI) procedures, practices, data and tools that are used by organization involved in EI activites in Europe.

Some of the questions will address the Epidemic Intelligence activites related to the MOOD diseases /disease systems specifically.

Instructions to complete this survey

In this survey, we will use the following definitions:

Epidemic intelligence (EI) is the early identification of potential health hazards related to outbreaks or incidents involving emerging infectious diseases that may represent a risk to the public/animal health, and their verification, assessment and investigation so that appropriate health control measures can be recommended. The EI includes the data streams from both event-based and indicator-based surveillance.

Event-based surveillance (EBS) is the capturing, filtering and verification of non-structured epidemiological data from a variety of informal sources (e.g., social media & electronic media news monitoring).

Indicator-based surveillance (IBS) is the collection, analysis and interpretation of verified and structured epidemiological data from routine surveillance sources (e.g., active and passive disease surveillance systems).

In this survey we use the definition of Epidemic Intelligence by Paquet et al. 2006

Why were you invited to participate in this survey

This survey is targeted for key gatekeepers in disease surveillance in public health, veterinary health-and One Health institutions in Europe. We have identified you as a gatekeeper in your institution and thus invited you to participate into this survey.

Your participation in this survey is entirely voluntary. You are not under any obligation to participate and you also have the right to refuse this invitation. If at any point in time during the survey you take the decision not to participate any further, you are free to do so immediately without any further discussion

Personal data protection

This survey will not collect personal information, except for your name, surname, position and country to ensure completeness of the survey. By participating to this survey, you agree that we use your data solely for the purposes of this study and no other exploitation of your personal information.

* I have understood and give my consent that my contributions will be processed and personal data is being kept for communication and acknowledgement purposes only.

- Yes, I wish to participate
- No, I need more information (Please contact the persons mentioned below)

Start the survey

This survey is in English language and it should take maximum of 60 minutes of your time.

Structure of the questionnaire:

1. Existence and organization of Epidemic intelligence.

- General question concerning EI activities within your institution

2. Three identical sections of disease surveillance with more detailed questions about EI activities.

- You will be provided a list of diseases in the beginning of each section.
- We ask you to choose diseases that are monitored within your institution by EI activities and describe their surveillance in details.
- Choose three diseases that you are most comfortable answering for.
- ***Please note: If your institution is involved with SARS-CoV-2 surveillance, we kindly ask you to choose it as one of the described diseases in the questionnaire.***

3. End of the questionnaire.

- Some additional questions and free space if you wish to clarify or comment something.

Additional information for the survey

- You can stop and resume the survey at any time, by making sure that you have ticked the box "*Save a backup on your local computer*" on top of this page. You might also save this survey as a draft and receive a link to finish completing it.
- Please note that the numbering of the questions depends on the answers you choose and therefore might not be logical.
- For further questions, please contact: Timothée Dub, *timothee.dub@thl.fi* and Henna Mäkelä, *henna.makela@thl.fi* at THL (Finnish Institute for Health and Welfare)

Existence and organization of Epidemic Intelligence (EI) activities

In this Section, please describe the general characteristics of the EI activities conducted in your institution for early detection, monitoring and assessment of signals of infectious disease emergence

Survey completed by:

* **1.** Name/surname

**The survey analysis will be conducted anonymously. Collection of your name is only required to contact you for further information and to acknowledge your participation in future publications and reports.*

2. Gender

* **3.** Position

4. Which of the following areas of preparedness and response are you working with?

- ☐ Management of epidemiological data
- ☐ Epidemic intelligence (EI)
- ☐ Risk assessment, response
- ☐ Planning, monitoring and evaluation
- ☐ Epidemic modelling
- ☐ Outbreak analytics
- ☐ Research and scientific writing
- ☐ Development and dissemination of information products
- ☐ Other

4.1. Please describe other activities

* **5.** Country

* **6.** Name of the institution

* **7.** Approximate number of staff in you institution

Provide the approximate number of the staff in numbers

* **8.** What is the scope of the institution? *multiple answers possible*

- ☐ Public health (PH)

- [ ] Veterinary health (VH)
- [ ] One health (OH)
- [ ] Other

8.1 If you chose "other", please describe

[ ]

* **9.** Which types of infectious diseases does your institution monitor?

- [ ] Human
- [ ] Animal
- [ ] Food products of animal origin
- [ ] Other

9.1. If you chose "other", please specify

[ ]

* **10.** Does your institution carry out Epidemic Intelligence activities?

If needed, see the description of Epidemic Intelligence on the starting page. By EI we mean both indicator- and event-based surveillance

- ( ) Yes
- ( ) No

10.1. Approximate number of Full Time Equivalents* currently involved in EI?

**A Full-Time Equivalent (FTE) is the amount of hours worked by an employee on a full time basis.*

*For example: In country X, where full-time basis is 40 hours/week, if 3 employees spend 20 hours per week on a project, then FTE is 3*20 hours /40 hours = 1.5 FTE.*

In country Y, where a full-time basis is 35 hours, if 2 employees work full time on a project, one employee works on it 20 hours a week and another one 15 hours a week, then FTE is (35 + 35 + 20 +15)/35 = 3 FTE.

- ( ) 1
- ( ) 2
- ( ) 3-5
- ( ) 5-10
- ( ) >10

10.2. Which structure(s) is responsible for conducting Epidemic Intelligence activities in your country?

Please provide the name of the structure and contact details of one of the responsible persons if known

[ ]

Please note: If your institution is not responsible for any EI activities, please go directly to last section "**End of the questionnaire**" and answer **questions 1 and 2**.

10.3. Is there a dedicated Epidemic Intelligence team in your institution?

- Yes
- No
- Other

10.3.1. What is the name of the service/unit/department or team?

10.3.2. If you chose "other", please describe

10.4. Are you personally conducting EI activities?

- Yes
- No

10.4.1. Please describe shortly which activities with EI you are conducting?

11. Does your institution have any standard operating procedures (SOPs) for EI?

- Yes
- No

11.1. Which aspects of EI these standard operating procedures are related to?
Multiple options possible

- [ ] Detection of signals of potential health hazards related to infectious disease outbreaks
- [ ] Filtering of these signals
- [ ] Verification of these signals
- [ ] Assessment of these signals
- [ ] Communication of these signals
- [ ] Other

11.1.2. If you chose "other", please describe

11.2. Are the procedure(s) publicly avaliable?

- Yes
- No

11.2.1. Could you provide the name/link to the website/document?

11.2.2. You can also upload your document here

The maximum file size is 1 MB

Before proceeding to next section, please make sure that questionnaire is finished loading dependencies

Disease 1

Please note: If your institution is involved with SARS-CoV-2 surveillance, we kindly ask you to choose it as one of the described diseases in the questionnaire.

1. Please pick a disease monitored within your institution by EI activities and describe their surveillance in details.

- Seasonal influenza (in humans)
- Highly pathogenic avian influenza (in animals)
- West Nile virus in humans
- West Nile virus in animals
- Chikungunya
- Dengue
- Zika
- Lyme borreliosis
- Tick-borne encephalitis
- Tularaemia
- Leptospirosis
- AMR in humans
- AMR in animals
- AMR in food products of animal origin
- SARS-CoV-2
- Disease X (i.e. disease threats of unknown origin)

Please note: Make sure that you will answer all the questions in this section based on activities with the disease you chose above.

Data sources for EI

In this section, please describe the data and sources you are monitoring as part of your EI activities, including the data and sources originating from both event-based and indicator-based surveillance.

2. What is the geographical scope of interest of the Epidemic Intelligence activities conducted in your institute regarding **this disease**?

Please choose one option from each row

Event-based surveillance (EBS) is the capturing, filtering and verification of non-structured epidemiological data from a variety of informal sources (e.g., social media & electronic media news monitoring).

Indicator-based surveillance (IBS) is the collection, analysis and interpretation of verified and structured epidemiological data from routine surveillance sources (e.g., active and passive disease surveillance systems).

	Indicator-based surveillance	Event-based surveillance	Both IBS and EBS	None
National	○	○	○	○
Bordering countries	○	○	○	○
Europe	○	○	○	○
Worldwide	○	○	○	○

Data sources for event-based (EBS) EI activities

3. For **this disease**, what are the sources of event-based surveillance (EBS) used to conduct EI activities in your institution?

Tick all that applies

Event-based surveillance (EBS) is the capturing, filtering and verification of non-structured epidemiological data from a variety of informal sources (e.g., social media & electronic media news monitoring).

	Scientific literature	Mainstream media (newspapers etc.)	Social media and /or blogs	Specialized internet sources (ProMED, Healthmap, gideon, etc.)	Official international notifications (WHO, EPIS)	Epidemic intelligence surveillance system (EIOS)
Sources for EBS	☐	☐	☐	☐	☐	☐

4. Are there other sources for **event-based surveillance**?

- ◯ Yes
- ◯ No

4.1. Please specify the other sources of information used for EB

Data sources for indicator-based (IBS) EI activities

5. What are the sources of indicator-based surveillance for **this disease** used to conduct EI activities in your institution?

Tick all that applies.

Indicator-based surveillance (IBS) is the collection, analysis and interpretation of verified and structured epidemiological data from routine surveillance sources (e.g., active and passive disease surveillance systems).

	Mandatory laboratory-based	Sentinel laboratory-based	Mandatory syndromic	Sentinel syndromic	Official public websites (WHO, OIE, ECDC)	Official international notifications (WHO, ADNS)	Other IBS sources (mortality monitoring etc.)
Sources for IBS	☐	☐	☐	☐	☐	☐	☐

6. Are there other sources for **indicator-based surveillance**?

- Yes
- No

6.1. Please specify the other sources of information used for IBS

Workflow for detection, verification and assessment of signals

This section will cover the detection and monitoring of signals of disease emergence

*Between EBS and IBS, the procedures for detection and monitoring of signals are likely to differ as a consequence of the origin of the data collected. Therefore, for **EBS** we refer to the **collection, filtering** and **verifying** of information i.e. signals of disease emergence. For **IBS** we refer to **the capturing, analysing and interpretation of information***

For signals from event-based sources

*By **collecting** information from event-based sources we mean the capture of new or updated information regarding potential health hazards related to outbreaks or incidents. This involves emerging infectious diseases through the screening of non-structured epidemiological data from a variety of sources such as:*

- *media reports*
- *national or international official sources (e.g. WHO, MoH)*
- *specialist aggregated websites (e.g. ProMED, HealthMap)*
- *notes and rumours (e.g. social media) by applying specific criteria.*

*By **filtering** the events we mean identifying potential health hazards related to outbreaks or incidents. This involves emerging infectious diseases considered relevant among the captured information.*

*By **verifying** captured information we mean verifying or validating the events that originate from unofficial sources: cross-checking with official and/or reliable media sources to ensure that the event detected is from a reputable source, real and fully understood.*

7. How does your institution collect, filter and verify information from the event-based sources for **this disease**?

- ☐ **Manually** (a fully human intervention based process for collection, filtering and verification)
- ☐ **Semi-automatically** (a combination of both automated processes and human intervention (E.g. for collection: automated information extraction from any of the above listed electronic sources (web-scraping); for filtering: supervised machine learning approaches to classify information according to relevance, etc; and human-based verifying: trained experts confirming the authenticity of information, manual screening of above listed sources)
- ☐ **Automatically** (a fully automated process for collection, filtering and verification of signals, without any human intervention (i.e. no manual processes involved).

7.1. Please shortly describe the process with emphasis on the tools and software used
*(i.e. for each of the three procedures (collection, filtering and verification) please list **methodology/ models used, software employed** etc.)*

Please note: This is an important question for us to understand the overall process. We kindly ask you to provide short overview of the process and tools used

For signals from indicator-based sources (IBS)

***Indicator-based surveillance (IBS)** is the collection, analysis and interpretation of verified and structured epidemiological data from routine surveillance sources (e.g., active and passive disease surveillance systems)*

*By **capturing** of indicators we mean the collection of indicator-based surveillance data for the early detection and monitoring of potential health hazards, which are related to outbreaks or incidents involving emerging infectious diseases.*

*By **analysis and interpretation** we mean assessing the significance of the data. E.g. through statistical comparison with baseline rates or thresholds to determine if the indicator is relevant and relates a potential health hazard.*

8. How does your institution capture, analyze and interpret information from the indicator-based sources for this disease?

- ☐ **Manually** (a fully human intervention based process for the collection, analysis and interpretation of data)

- ☐ **Semi-automatically** (a combination of both automated processes and human intervention (e.g. for collection: routine automated collection of indicators from health data; for analysis: automatic detection of clusters, patterns or trends in data using statistical methodology; generation of automatic alerts using estimated pre-defined thresholds; or routine model-based epidemic forecasting, etc; followed by human-based interpretation: trained experts assessing whether an alert may arise from artefacts in the data)
- ☐ **Automatically** (a fully automated process for the collection, analyses and interpretation of signals, without any human intervention (i.e. no manual processes involved)

8.1. Please shortly describe the process with emphasis on the tools and software used *(i.e. for each of the three procedures, please list **methodology/ models used, software employed** etc.)*

Please note: This is an important question for us to understand the overall process. We kindly ask you to provide short overview of the process and tools used

Assessment of signals (information from both EBS and IBS)

9. How does your institution define a signal requiring assessment regarding **this disease**?

*The **assessment** is made, following analysis and interpretation of both IBS and EBS signals, to estimate the risk associated with the signal that has been detected.*

	Semi-automatically	Manually: Expert review of signal
Assessment done:	○	○

9.1. Can you describe the process with **this disease**? What are the processes leading to action or further investigation?

Communication of alerts

This section covers communication of public health threats from a general perspective. When signals of potential infectious diseases related health hazards have been detected, how is information transmitted and to whom?

10. What is the destination of alerts with **this disease**?

- ☐ Communication to the **general public**
- ☐ **Restricted-access** communication on a **national level** *(i.e. communication between public health and animal health, communication between regional surveillance officers)*

☐ **Restricted-access** communication on an international level

10.1. Communication to the general public: Could you briefly specify to whom and how the signals are communicated?

10.2. Restricted-access communication on a national level: Could you briefly specify to whom and how the signals are communicated?

10.3. Restricted-access communication in an international level: Could you briefly specify to whom and how the signals are communicated?

International EI collaboration

The following section will address the existence international collaboration and the cross-collaboration of the EI systems.

11. Is there any collaboration with neighbouring countries or international structures (e.g., WHO, FAO, ECDC) regarding **this disease**?

Collaboration on the level of e.g. surveillance design, data collection, data sharing, data analyses, data management etc.

- ○ Yes
- ○ No

11.1. In which of the following sections does the collaboration take place? *Tick all that applies*

	Surveillance design	Data collection	Data sharing	Sharing of surveillance results	Data management or/and storage	Data analysis and interpretation	Communication
International structures	☐	☐	☐	☐	☐	☐	☐
Neighbouring countries	☐	☐	☐	☐	☐	☐	☐

11.2. Please shortly describe the level of international collaboration.(e.g. Reporting to TESSy, active collaboration etc.)

One health, public health and veterinary health EI collaboration

The following section will address the existence of one health, including public health & veterinary health collaboration and the cross-collaboration of the EI systems

12. Is there collaboration with another health sector as part of the EI processes nationally regarding **this disease**?

- Yes
- No

12.1. In which of the following sections does the collaboration take place? *Tick all that applies*

	Surveillance design	Data collection	Data sharing	Sharing of surveillance results	Data management or/and storage	Data analysis and interpretation	Communication
National collaboration with other health sectors	☐	☐	☐	☐	☐	☐	☐

12.2. Which health sectors/other structures are you collaborating with?

What is the level of collaboration regarding **surveillance design**?

- ☐ Undertaken by a single sector for all surveillance components
- ☐ Cross-sectoral consultation but undertaken separately in each sectors
- ☐ Undertaken by a multi-sectoral working group
- ☐ Undertaken by a multi-sectoral body

What is the level of collaboration regarding **data collection**?

- ☐ Undertaken by a single sector for all surveillance components
- ☐ Harmonized across sector
- ☐ Joint activities across sectors

What is the level of collaboration regarding **data sharing**?

- ☐ Only notification of unusual events
- ☐ Ongoing data exchange

What is the level of collaboration regarding **sharing of surveillance results**?

- ☐ Only notification of unusual events
- ☐ Ongoing sharing of surveillance indicators

What is the level of collaboration regarding **data management or/and storage**?

- ☐ Undertaken by a single sector for all surveillance components
- ☐ Harmonized across sector
- ☐ Joint activities across sectors

What is the level of collaboration regarding **data analysis and interpretation**?

- ☐ Undertaken separately and then compared by a single sector
- ☐ Jointly undertaken by a single sector for all components
- ☐ Undertaken separately and then compared by a multi-sectoral working group
- ☐ Jointly undertaken by a multi-sectoral working group or body

What is the level of collaboration regarding **communication**?

- ☐ Communication is public
- ☐ Communication concerning surveillance actors
- ☐ Communication is concerning beneficiaries

Before proceeding to next section, please make sure that questionnaire is finished loading dependencies

Disease 2

1. Please pick a disease monitored within your institution by EI activities and describe their surveillance in details.

- Seasonal influenza (in humans)
- Highly pathogenic avian influenza (in animals)
- West Nile virus in humans
- West Nile virus in animals
- Chikungunya
- Dengue
- Zika
- Lyme borreliosis
- Tick-borne encephalitis
- Tularaemia
- Leptospirosis
- AMR in humans
- AMR in animals
- AMR in food products of animal origin
- SARS-CoV-2
- Disease X (i.e. disease threats of unknown origin)

Please note: Make sure that you will answer all the questions in this section based on activities with the disease you chose above.

Data sources for EI

In this section, please describe the data and sources you are monitoring as part of your EI activities, including the data and sources originating from both event-based and indicator-based surveillance.

2. What is the geographical scope of interest of the Epidemic Intelligence activities conducted in your institute regarding **this disease**?

Please choose one option from each row

Event-based surveillance (EBS) is the capturing, filtering and verification of non-structured epidemiological data from a variety of informal sources (e.g., social media & electronic media news monitoring).

Indicator-based surveillance (IBS) is the collection, analysis and interpretation of verified and structured epidemiological data from routine surveillance sources (e.g., active and passive disease surveillance systems).

	Indicator-based surveillance	Event-based surveillance	Both IBS and EBS	None
National				
Bordering countries				

Europe	○	○	○	○
Worldwide	○	○	○	○

Data sources for event-based (EBS) EI activities

3. For **this disease**, what are the sources of event-based surveillance (EBS) used to conduct EI activities in your institution?

Tick all that applies

Event-based surveillance (EBS) is the capturing, filtering and verification of non-structured epidemiological data from a variety of informal sources (e.g., social media & electronic media news monitoring).

	Scientific literature	Mainstream media (newspapers etc.)	Social media and /or blogs	Specialized internet sources (ProMED, Healthmap, gideon, etc.)	Official international notifications (WHO, EPIS)	Epidemic intelligence surveillance system (EIOS)
Sources for EBS	☐	☐	☐	☐	☐	☐

4. Are there other sources for **event-based surveillance**?

- Yes
- No

4.1. Please specify the other sources of information used for EBS

Data sources for indicator-based (IBS) EI activities

5. What are the sources of indicator-based surveillance for **this disease** used to conduct EI activities in your institution?

Tick all that applies.

Indicator-based surveillance (IBS) is the collection, analysis and interpretation of verified and structured epidemiological data from routine surveillance sources (e.g., active and passive disease surveillance systems).

	Mandatory laboratory-based	Sentinel laboratory-based	Mandatory syndromic	Sentinel syndromic	Official public websites (WHO, OIE, ECDC)	Official international notifications (WHO, ADNS)	Other IBS sources (mortality monitoring etc.)
Sources for IBS	☐	☐	☐	☐	☐	☐	☐

6. Are there other sources for **indicator-based surveillance**?

- Yes
- No

6.1. Please specify the other sources of information used for IBS

Workflow for detection, verification and assessment of signals

This section will cover the detection and monitoring of signals of disease emergence

*Between EBS and IBS, the procedures for detection and monitoring of signals are likely to differ as a consequence of the origin of the data collected. Therefore, for **EBS** we refer to the **collection, filtering** and **verifying** of information i.e. signals of disease emergence. For **IBS** we refer to **the capturing, analysing and interpretation of information***

For signals from event-based sources

*By **collecting** information from event-based sources we mean the capture of new or updated information regarding potential health hazards related to outbreaks or incidents. This involves emerging infectious diseases through the screening of non-structured epidemiological data from a variety of sources such as:*

- *media reports*
- *national or international official sources (e.g. WHO, MoH)*
- *specialist aggregated websites (e.g. ProMED, HealthMap)*
- *notes and rumours (e.g. social media) by applying specific criteria.*

*By **filtering** the events we mean identifying potential health hazards related to outbreaks or incidents. This involves emerging infectious diseases considered relevant among the captured information.*

*By **verifying** captured information we mean verifying or validating the events that originate from unofficial sources: cross-checking with official and/or reliable media sources to ensure that the event detected is from a reputable source, real and fully understood.*

7. How does your institution collect, filter and verify information from the event-based sources for **this disease**?

- ☐ **Manually** (a fully human intervention based process for collection, filtering and verification)
- ☐ **Semi-automatically** (a combination of both automated processes and human intervention (E.g. for collection: automated information extraction from any of the above listed electronic sources (web-scraping); for filtering: supervised machine learning approaches to classify information according to relevance, etc; and human-based verifying: trained experts confirming the authenticity of information, manual screening of above listed sources)
- ☐ **Automatically** (a fully automated process for collection, filtering and verification of signals, without any human intervention (i.e. no manual processes involved).

7.1. Please shortly describe the process with emphasis on the tools and software used
*(i.e. for each of the three procedures (collection, filtering and verification), please list **methodology/ models used, software employed** etc.)*

Please note: This is an important question for us to understand the overall process. We kindly ask you to provide short overview of the process and tools used

For signals from <u>indicator-based</u> sources (IBS)

***Indicator-based surveillance (IBS)** is the collection, analysis and interpretation of verified and structured epidemiological data from routine surveillance sources (e.g., active and passive disease surveillance systems)*

*By **capturing** of indicators we mean the collection of indicator-based surveillance data for the early detection and monitoring of potential health hazards, which are related to outbreaks or incidents involving emerging infectious diseases.*

*By **analysis and interpretation** we mean assessing the significance of the data. E.g. through statistical comparison with baseline rates or thresholds to determine if the indicator is relevant and relates a potential health hazard.*

8. How does your institution capture, analyze and interpret information from the indicator-based sources for this disease?

- ☐ **Manually** (a fully human intervention based process for the collection, analysis and interpretation of data)

- ☐ **Semi-automatically** (a combination of both automated processes and human intervention (e.g. for collection: routine automated collection of indicators from health data; for analysis: automatic detection of clusters, patterns or trends in data using statistical methodology; generation of automatic alerts using estimated pre-defined thresholds; or routine model-based epidemic forecasting, etc; followed by human-based interpretation: trained experts assessing whether an alert may arise from artefacts in the data)
- ☐ **Automatically** (a fully automated process for the collection, analyses and interpretation of signals, without any human intervention (i.e. no manual processes involved)

8.1. Please shortly describe the process with emphasis on the tools and software used *(i.e. for each of the three procedures, please list **methodology/ models used, software employed** etc.)*

Please note: This is an important question for us to understand the overall process. We kindly ask you to provide short overview of the process and tools used

Assessment of signals (information from both EBS and IBS)

9. How does your institution define a signal requiring assessment regarding **this disease**?

*The **assessment** is made, following analysis and interpretation of both IBS and EBS signals, to estimate the risk associated with the signal that has been detected.*

	Semi-automatically	Manually: Expert review of signal
Assessment done:	○	○

9.1. Can you describe the process with **this disease**? What are the processes leading to action or further investigation?

Communication of alerts

This section covers communication of public health threats from a general perspective. When signals of potential infectious diseases related health hazards have been detected, how is information transmitted and to whom?

10. What is the destination of alerts with **this disease**?

- ☐ Communication to the **general public**
- ☐ **Restricted-access** communication on a **national level** *(i.e. communication between public health and animal health, communication between regional surveillance officers)*

☐ **Restricted-access** communication on an international level

10.1. Communication to the general public: Could you briefly specify to whom and how the signals are communicated?

10.2. Restricted-access communication on a national level: Could you briefly specify to whom and how the signals are communicated?

10.3. Restricted-access communication in an international level: Could you briefly specify to whom and how the signals are communicated?

International EI collaboration

The following section will address the existence international collaboration and the cross-collaboration of the EI systems.

11. Is there any collaboration with neighbouring countries or international structures (e.g., WHO, FAO, ECDC) regarding **this disease**?

Collaboration on the level of e.g. surveillance design, data collection, data sharing, data analyses, data management etc.

- ◯ Yes
- ◯ No

11.1. In which of the following sections does the collaboration take place? *Tick all that applies*

	Surveillance design	Data collection	Data sharing	Sharing of surveillance results	Data management or/and storage	Data analysis and interpretation	Communication
International structures	☐	☐	☐	☐	☐	☐	☐
Neighbouring countries	☐	☐	☐	☐	☐	☐	☐

11.2. Please shortly describe the level of international collaboration.(e.g. Reporting to TESSy, active collaboration etc.)

One health, public health and veterinary health EI collaboration

The following section will address the existence of one health, including public health & veterinary health collaboration and the cross-collaboration of the EI systems.

12. Is there collaboration with another health sector as part of the EI processes nationally regarding **this disease**?

- Yes
- No

12.1. In which of the following sections does the collaboration take place? *Tick all that applies*

	Surveillance design	Data collection	Data sharing	Sharing of surveillance results	Data management or/and storage	Data analysis and interpretation	Communication
National collaboration with other health sectors	☐	☐	☐	☐	☐	☐	☐

12.2. Which health sectors/other structures are you collaborating with?

What is the level of collaboration regarding **surveillance design**?

- ☐ Undertaken by a single sector for all surveillance components
- ☐ Cross-sectoral consultation but undertaken separately in each sectors
- ☐ Undertaken by a multi-sectoral working group
- ☐ Undertaken by a multi-sectoral body

What is the level of collaboration regarding **data collection**?

- ☐ Undertaken by a single sector for all surveillance components
- ☐ Harmonized across sector
- ☐ Joint activities across sectors

What is the level of collaboration regarding **data sharing**?

- ☐ Only notification of unusual events
- ☐ Ongoing data exchange

What is the level of collaboration regarding **sharing of surveillance results**?

- ☐ Only notification of unusual events
- ☐ Ongoing sharing of surveillance indicators

What is the level of collaboration regarding **data management or/and storage**?

- ☐ Undertaken by a single sector for all surveillance components
- ☐ Harmonized across sector
- ☐ Joint activities across sectors

What is the level of collaboration regarding **data analysis and interpretation**?

- ☐ Undertaken separately and then compared by a single sector
- ☐ Jointly undertaken by a single sector for all components
- ☐ Undertaken separately and then compared by a multi-sectoral working group
- ☐ Jointly undertaken by a multi-sectoral working group or body

What is the level of collaboration regarding **communication**?

- ☐ Communication is public
- ☐ Communication concerning surveillance actors
- ☐ Communication is concerning beneficiaries

Before proceeding to next section, please make sure that questionnaire is finished loading dependencies

Disease 3

1. Please pick a disease monitored within your institution by EI activities and describe their surveillance in details.

- Seasonal influenza (in humans)
- Highly pathogenic avian influenza (in animals)
- West Nile virus in humans
- West Nile virus in animals
- Chikungunya
- Dengue
- Zika
- Lyme borreliosis
- Tick-borne encephalitis
- Tularaemia
- Leptospirosis
- AMR in humans
- AMR in animals
- AMR in food products of animal origin
- SARS-CoV-2
- Disease X (i.e. disease threats of unknown origin)

Please note: Make sure that you will answer all the questions in this section based on activities with the disease you chose above.

Data sources for EI

In this section, please describe the data and sources you are monitoring as part of your EI activities, including the data and sources originating from both event-based and indicator-based surveillance.

2. What is the geographical scope of interest of the Epidemic Intelligence activities conducted in your institute regarding **this disease**?

Please choose one option from each row

Event-based surveillance (EBS) is the capturing, filtering and verification of non-structured epidemiological data from a variety of informal sources (e.g., social media & electronic media news monitoring).

Indicator-based surveillance (IBS) is the collection, analysis and interpretation of verified and structured epidemiological data from routine surveillance sources (e.g., active and passive disease surveillance systems).

	Indicator-based surveillance	Event-based surveillance	Both IBS and EBS	None
National	○	○	○	○
Bordering countries	○	○	○	○

Europe	○	○	○	○
Worldwide	○	○	○	○

Data sources for event-based (EBS) EI activities

3. For **this disease**, what are the sources of event-based surveillance (EBS) used to conduct EI activities in your institution?

Tick all that applies

Event-based surveillance (EBS) is the capturing, filtering and verification of non-structured epidemiological data from a variety of informal sources (e.g., social media & electronic media news monitoring).

	Scientific literature	Mainstream media (newspapers etc.)	Social media and /or blogs	Specialized internet sources (ProMED, Healthmap, gideon, etc.)	Official international notifications (WHO, EPIS)	Epidemic intelligence surveillance system (EIOS)
Sources for EBS	☐	☐	☐	☐	☐	☐

4. Are there other sources for **event-based surveillance**?

- Yes
- No

4.1. Please specify the other sources of information used for EBS

Data sources for indicator-based (IBS) EI activities

5. What are the sources of indicator-based surveillance for **this disease** used to conduct EI activities in your institution?

Tick all that applies.

Indicator-based surveillance (IBS) is the collection, analysis and interpretation of verified and structured epidemiological data from routine surveillance sources (e.g., active and passive disease surveillance systems).

	Mandatory laboratory-based	Sentinel laboratory-based	Mandatory syndromic	Sentinel syndromic	Official public websites (WHO, OIE, ECDC)	Official international notifications (WHO, ADNS)	Other IBS sources (mortality monitoring etc.)
Sources for IBS	☐	☐	☐	☐	☐	☐	☐

6. Are there other sources for **indicator-based surveillance**?

- Yes
- No

6.1. Please specify the other sources of information used for IBS

Workflow for detection, verification and assessment of signals

This section will cover the detection and monitoring of signals of disease emergence

*Between EBS and IBS, the procedures for detection and monitoring of signals are likely to differ as a consequence of the origin of the data collected. Therefore, for **EBS** we refer to the **collection, filtering** and **verifying** of information i.e. signals of disease emergence. For **IBS** we refer to **the capturing, analysing and interpretation of information***

For signals from event-based sources

*By **collecting** information from event-based sources we mean the capture of new or updated information regarding potential health hazards related to outbreaks or incidents. This involves emerging infectious diseases through the screening of non-structured epidemiological data from a variety of sources such as:*

- *media reports*
- *national or international official sources (e.g. WHO, MoH)*
- *specialist aggregated websites (e.g. ProMED, HealthMap)*
- *notes and rumours (e.g. social media) by applying specific criteria.*

*By **filtering** the events we mean identifying potential health hazards related to outbreaks or incidents. This involves emerging infectious diseases considered relevant among the captured information.*

*By **verifying** captured information we mean verifying or validating the events that originate from unofficial sources: cross-checking with official and/or reliable media sources to ensure that the event detected is from a reputable source, real and fully understood.*

7. How does your institution collect, filter and verify information from the event-based sources for **this disease**?

- ☐ **Manually** (a fully human intervention based process for collection, filtering and verification)
- ☐ **Semi-automatically** (a combination of both automated processes and human intervention (E.g. for collection: automated information extraction from any of the above listed electronic sources (web-scraping); for filtering: supervised machine learning approaches to classify information according to relevance, etc; and human-based verifying: trained experts confirming the authenticity of information, manual screening of above listed sources)
- ☐ **Automatically** (a fully automated process for collection, filtering and verification of signals, without any human intervention (i.e. no manual processes involved).

7.1. Please shortly describe the process with emphasis on the tools and software used
*(i.e. for each of the three procedures (collection, filtering and verification), please list **methodology/ models used, software employed** etc.)*

Please note: This is an important question for us to understand the overall process. We kindly ask you to provide short overview of the process and tools used

For signals from indicator-based sources (IBS)

***Indicator-based surveillance (IBS)** is the collection, analysis and interpretation of verified and structured epidemiological data from routine surveillance sources (e.g., active and passive disease surveillance systems)*

*By **capturing** of indicators we mean the collection of indicator-based surveillance data for the early detection and monitoring of potential health hazards, which are related to outbreaks or incidents involving emerging infectious diseases.*

*By **analysis and interpretation** we mean assessing the significance of the data. E.g. through statistical comparison with baseline rates or thresholds to determine if the indicator is relevant and relates a potential health hazard.*

8. How does your institution capture, analyze and interpret information from the indicator-based sources for this disease?

- ☐ **Manually** (a fully human intervention based process for the collection, analysis and interpretation of data)

- ☐ **Semi-automatically** (a combination of both automated processes and human intervention (e.g. for collection: routine automated collection of indicators from health data; for analysis: automatic detection of clusters, patterns or trends in data using statistical methodology; generation of automatic alerts using estimated pre-defined thresholds; or routine model-based epidemic forecasting, etc; followed by human-based interpretation: trained experts assessing whether an alert may arise from artefacts in the data)
- ☐ **Automatically** (a fully automated process for the collection, analyses and interpretation of signals, without any human intervention (i.e. no manual processes involved)

8.1. Please shortly describe the process with emphasis on the tools and software used *(i.e. for each of the three procedures, please list **methodology/ models used, software employed** etc.)*

Please note: This is an important question for us to understand the overall process. We kindly ask you to provide short overview of the process and tools used

Assessment of signals (information from both EBS and IBS)

9. How does your institution define a signal requiring assessment regarding **this disease**?

*The **assessment** is made, following analysis and interpretation of both IBS and EBS signals, to estimate the risk associated with the signal that has been detected.*

	Semi-automatically	Manually: Expert review of signal
Assessment done:	○	○

9.1. Can you describe the process with **this disease**? What are the processes leading to action or further investigation?

Communication of alerts

This section covers communication of public health threats from a general perspective. When signals of potential infectious diseases related health hazards have been detected, how is information transmitted and to whom?

10. What is the destination of alerts with **this disease**?

- ☐ Communication to the **general public**
- ☐ **Restricted-access** communication on a **national level** *(i.e. communication between public health and animal health, communication between regional surveillance officers)*

☐ **Restricted-access** communication on an international level

10.1. Communication to the general public: Could you briefly specify to whom and how the signals are communicated?

10.2. Restricted-access communication on a national level: Could you briefly specify to whom and how the signals are communicated?

10.3. Restricted-access communication in an international level: Could you briefly specify to whom and how the signals are communicated?

International EI collaboration

The following section will address the existence international collaboration and the cross-collaboration of the EI systems.

11. Is there any collaboration with neighbouring countries or international structures (e.g., WHO, FAO, ECDC) regarding **this disease**?

Collaboration on the level of e.g. surveillance design, data collection, data sharing, data analyses, data management etc.

- ○ Yes
- ○ No

11.1. In which of the following sections does the collaboration take place? *Tick all that applies*

	Surveillance design	Data collection	Data sharing	Sharing of surveillance results	Data management or/and storage	Data analysis and interpretation	Communication
International structures	☐	☐	☐	☐	☐	☐	☐
Neighbouring countries	☐	☐	☐	☐	☐	☐	☐

11.2. Please shortly describe the level of international collaboration.(e.g. Reporting to TESSy, active collaboration etc.)

One health, public health and veterinary health EI collaboration

The following section will address the existence of one health, including public health & veterinary health collaboration and the cross-collaboration of the EI systems.

12. Is there collaboration with another health sector as part of the EI processes nationally regarding **this disease**?

- Yes
- No

12.1. In which of the following sections does the collaboration take place? *Tick all that applies*

	Surveillance design	Data collection	Data sharing	Sharing of surveillance results	Data management or/and storage	Data analysis and interpretation	Communication
National collaboration with other health sectors	☐	☐	☐	☐	☐	☐	☐

12.2. Which health sectors/other structures are you collaborating with?

What is the level of collaboration regarding **surveillance design**?

- ☐ Undertaken by a single sector for all surveillance components
- ☐ Cross-sectoral consultation but undertaken separately in each sectors
- ☐ Undertaken by a multi-sectoral working group
- ☐ Undertaken by a multi-sectoral body

What is the level of collaboration regarding **data collection**?

- ☐ Undertaken by a single sector for all surveillance components
- ☐ Harmonized across sector
- ☐ Joint activities across sectors

What is the level of collaboration regarding **data sharing**?

- ☐ Only notification of unusual events
- ☐ Ongoing data exchange

What is the level of collaboration regarding **sharing of surveillance results**?

- ☐ Only notification of unusual events
- ☐ Ongoing sharing of surveillance indicators

What is the level of collaboration regarding **data management or/and storage**?

- ☐ Undertaken by a single sector for all surveillance components
- ☐ Harmonized across sector
- ☐ Joint activities across sectors

What is the level of collaboration regarding **data analysis and interpretation**?

- ☐ Undertaken separately and then compared by a single sector
- ☐ Jointly undertaken by a single sector for all components
- ☐ Undertaken separately and then compared by a multi-sectoral working group
- ☐ Jointly undertaken by a multi-sectoral working group or body

What is the level of collaboration regarding **communication**?

- ☐ Communication is public
- ☐ Communication concerning surveillance actors
- ☐ Communication is concerning beneficiaries

Before proceeding to next section, please make sure that questionnaire is finished loading dependencies

End of the questionnaire

1. Monitoring of MOOD model diseases

If you have already described this disease in the previous section, please mark the first column with an X.

Please provide one answers per each row

	Described earlier	Monitored by your institution.<br>-**If surveillance is identical to any disease you have described earlier, please mark the name of the disease**<br>**-If not, please give <u>name and email address</u> of the person responsible of the surveillance we can contact**	Monitored by another institution.<br>**Please provide the name of the institution and if possible details of contact person responsible**	Not monitored in your country<br>**(mark with X)**
Seasonal influenza (in humans)				
Zoonotic influenza (in animals)				
Leptospirosis				
AMR in humans				
AMR in animals				
AMR in food products of animal origin				
SARS-CoV-2				
Disease X (i.e. disease threats of unknown origin)				

2. Monitoring of MOOD model diseases

If you have already described this disease in the previous section, please mark the first column with an X.

Please provide one answer per each row

	Described earlier	Monitored by your institution.<br>-**If surveillance is identical to any disease you have described earlier, please mark the name of the disease**<br>**-If not, please give name and email address of the person responsible of the surveillance we can contact**	Monitored by another institution.<br>**Please provide the name of the institution and if possible details of contact person responsible**	Not monitored in your country<br>**(mark with X)**
West Nile virus in humans				
West Nile virus in animals				
Chikungunya				
Dengue				
Zika				
Lyme borreliosis				
Tick-borne encephalitis				
Tularaemia				

3. Are there other diseases that are carefully monitored through your EI activities that were not mentioned in this survey?

- Yes
- No

3.1. What disease/which diseases? Could you provide the name and email of the person/persons responsible?

4. What are the main challenges of the EI system in your institution?

If the current SARS-CoV-2 outbreak revealed any particular existing gaps and challenges related to the early detection and monitoring of future disease X, we would be very interested to hear about them

5. Which tools, services, knowledge or other outputs and outcomes would you like your institutions´ EI system to have?

Acknowledgements

* **6.** Do you agree your name, surname and institution to be cited in the acknowledgements of the eventual reports and publications?

- Yes
- No

* **7.** Do you agree to be contacted for further questions regarding this survey?

- Yes
- No

8. Is there is something that you wish to clarify or comment?

For further questions, please contact: Timothée Dub, THL, Finland at: timothee.dub@thl.fi or Henna Mäkelä, Finland THL at: henna.makela@thl.fi

Thank you for your time and participating to this survey!
