# Supplementary material for: Epidemic intelligence activities among national public and animal health agencies: a European cross-sectional study
Source: BMC Public Health. 2023 Aug 4;23:1488. doi: 10.1186/s12889-023-16396-y (PMC10401758; doi:10.1186/s12889-023-16396-y)
Supplement: Supplementary file 2 — Additional file 2: Supplementary Table 1. Disease specific epidemic intelligence activities described per countries. * Responses provided by 2 gatekeeping agencies. Supplementary Table 2. Characteristics of respondents per diseases described. * Non mutually exclusive. [file 12889_2023_16396_MOESM2_ESM.docx]

## Supplementary table 1: Disease specific epidemic intelligence activities described per countries

|  | **HPAI in animals (n=17)** | **Seasonal influenza (n=10)** | **WNV in animals (n=7)** | **WNV in humans (n=9)** | **Leptospirosis and Tularemia in animals (n=7)** | **Invasive MBD in humans (n=6)** | **AMR in animals (n=6)** | **SARS-COV-2 in humans (n=12)** | **SARS-CoV-2 in animals (n=8)** |
| --- | --- | --- | --- | --- | --- | --- | --- | --- | --- |
| **Albania** | X | X |  |  |  |  |  |  | X |
| **Belgium** |  | X |  | X |  |  |  | X |  |
| **Denmark** | X* | X | X |  |  |  |  | X | X |
| **Finland** | X | X |  |  | X |  |  | X |  |
| **France** | X |  | X | X |  | X |  | X | X |
| **Germany** |  |  |  |  |  |  |  | X |  |
| **Great Britain (England, Wales, Scotland)** |  |  |  |  | X |  | X |  |  |
| **Hungary** | X |  | X |  | X |  |  |  |  |
| **Iceland** | X |  |  |  |  |  |  |  |  |
| **Ireland** | X |  |  |  | X |  |  |  |  |
| **Italy** | X |  | X | X |  | X |  | X | X |
| **Kosovo** |  | X |  | X |  |  |  |  |  |
| **Lithuania** |  | X |  |  |  | X |  | X |  |
| **Montenegro** |  |  |  | X |  | X |  | X |  |
| **Northern Ireland** | X | X |  |  |  |  | X | X |  |
| **Norway** | X |  |  |  |  |  | X |  | X |
| **Serbia** |  |  |  | X |  | X |  |  |  |
| **Slovakia** | X |  | X |  | X |  |  |  | X |
| **Slovenia** | X | X |  | X |  |  |  | X |  |
| **Spain** | X |  | X | X |  | X | X |  |  |
| **Sweden** | X |  |  |  | X |  |  |  | X |
| **Switzerland** | X |  | X |  | X |  | X |  |  |
| **The Netherlands** | X | X |  | X |  |  | X | X | X |
| **Wales** |  | X |  |  |  |  |  | X |  |

* Responses provided by 2 gatekeeping agencies

## Supplementary table 2: Characteristics of respondents per diseases described

|  | **HPAI in animals (n=17)** | **Seasonal influenza (n=10)** | **WNV in animals (n=7)** | **WNV in humans (N=9)** | **Leptospirosis and Tularemia in animals (n=7)** | **Invasive MBD in humans (n=6)** | **AMR in animals (n=6)** | **SARS-COV-2 in humans (n=12)** | **SARS-CoV-2 in animals (n=8)** |
| --- | --- | --- | --- | --- | --- | --- | --- | --- | --- |
| **Mandate of the institute*** | | | | | | | | | |
| One Health | 12 (71%) | 4 (40%) | 5 (71%) | 2 (22%) | 5 (71%) | 1 (17%) | 6 (100%) | 5 (42%) | 5 (63%) |
| Public health | 5 (29%) | 10 (100%) | 6 (86%) | 9 (100%) | 0 | 6 (100%) | 1 (17%) | 12 (100%) | 2 (25%) |
| Animal health | 15 (89%) | 2 (20%) | 6 (86%) | 2 (22%) | 6 (85%) | 1 (17%) | 6 (100%) | 3 (25%) | 7 (88%) |
| Other | 3 (18%) | 0 | 2 (29%) | 2 (22%) | 2 (29%) | 2 (33%) | 1 (17%) | 1 (8%) | 1 (13%) |
| **Diseases monitored*** | | | | | | | | | |
| Human infectious diseases | 3 (18%) | 10 (100%) | 1 (14%) | 9 (100%) | 0 | 6 (100%) | 0 | 12 (100%) | 2 (25%) |
| Animal infectious diseases | 17 (100%) | 4 (40%) | 7 (100%) | 3 (33%%) | 7 (100%) | 1 (17%) | 6 (100%) | 4 (33%) | 8 (100%) |
| Food products of animal origin | 11 (65%) | 3 (30%) | 3 (43%) | 4 (44%) | 3 (43%) | 2 (33%) | 3 (50%) | 4 (33%) | 3 (38%) |

* Non mutually exclusive
